# A New Process for Measuring the Solubility of Salicylic Acid

**Published:** 1894-01

**Authors:** 


A NEW PROCESS FOR INCREASING THE SOLUBILITY OF SALICYLIC ACID. (*Bollettino formaceutico*, Rome and Milan, 1893, p. 3.) By Carcarro and Cesaris.

Salicylic acid has been recommended as a substitute for bichloride of mercury in surgical and gynæcological practice, on account of the highly poisonous character of the mercurial salt; but of the very slight solubility of salicylic acid has been an obstacle to its employment for this purpose, the authors submit the following formula:

| | |
|---|---|
| Boracic acid | 12 parts. |
| Salicylic acid | 2 " |
| Water | 1000 " |

By heating this mixture a solutian is rapidly obtained, which does not form any precipitate on cooling.—*Inter. Med. Mag.*

